# Pathways to Green Perspectives: Production and Characterization of Polylactide (PLA) Nanocomposites Filled with Superparamagnetic Magnetite Nanoparticles

**DOI:** 10.3390/ma14185154

**Published:** 2021-09-08

**Authors:** Marius Murariu, Armando Galluzzi, Yoann Paint, Oltea Murariu, Jean-Marie Raquez, Massimiliano Polichetti, Philippe Dubois

**Affiliations:** 1 Laboratory of Polymeric and Composite Materials, Materia Nova Materials R&D Center & UMons Innovation Center, 3 Avenue Copernic, 7000 Mons, Belgium; yoann.paint@materianova.be (Y.P.); oltea.murariu@materianova.be (O.M.); 2 Department of Physics E.R. Caianiello, University of Salerno, and CNR-SPIN (Salerno), via Giovanni Paolo II, 84084 Fisciano, Italy; agalluzzi@unisa.it (A.G.); mpolichetti@unisa.it (M.P.); 3 Laboratory of Polymeric and Composite Materials (LPCM), Center of Innovation and Research in Materials and Polymers (CIRMAP), University of Mons (UMONS), 7000 Mons, Belgium; jean-marie.raquez@umons.ac.be

**Keywords:** biopolymers, poly(lactic acid), PLA, nanocomposites, magnetite (Fe_3_O_4_), reactive surface treatment, melt-compounding, thermal and morphology characterizations, superparamagnetic properties

## Abstract

In the category of biopolymers, polylactide or polylactic acid (PLA) is one of the most promising candidates considered for future developments, as it is not only biodegradable under industrial composting conditions, but it is produced from renewable natural resources. The modification of PLA through the addition of nanofillers is considered as a modern approach to improve its main characteristic features (mechanical, thermal, barrier, etc.) and to obtain specific end-use properties. Iron oxide nanoparticles (NPs) of low dimension (10–20 nm) such as magnetite (Fe_3_O_4_), exhibit strong magnetization in magnetic field, are biocompatible and show low toxicity, and can be considered in the production of polymer nanocomposites requiring superparamagnetic properties. Accordingly, PLA was mixed by melt-compounding with 4–16 wt.% magnetite NPs. Surface treatment of NPs with a reactive polymethylhydrogensiloxane (MHX) was investigated to render the nanofiller water repellent, less sensitive to moisture and to reduce the catalytic effects at high temperature of iron (from magnetite) on PLA macromolecular chains. The characterization of nanocomposites was focused on the differences of the rheology and morphology, modification, and improvements in the thermal properties using surface treated NPs, while the superparamagnetic behavior was confirmed by VSM (vibrating sample magnetometer) measurements. The PLA−magnetite nanocomposites had strong magnetization properties at low magnetic field (values close to 70% of M_max_ at H = 0.2 T), while the maximum magnetic signal (M_max_) was mainly determined by the loading of the nanofiller, without any significant differences linked to the surface treatment of MNPs. These bionanocomposites showing superparamagnetic properties, close to zero magnetic remanence, and coercivity, can be further produced at a larger scale by melt-compounding and can be designed for special end-use applications, going from biomedical to technical areas.

## 1. Introduction

The tremendous interest in the production of materials based on biopolymers is connected to the important demands received from consumers for more environmentally sustainable products as well as from the amplified restrictions for the utilization of polymers with a high “carbon footprint” of a petrochemical origin, particularly in applications such as packaging, automotive, electrical, and electronics, among others [1,2]. Accordingly, one of the main objectives is to replace “fossil carbon” with “renewable carbon”. Moreover, the biodegradability of polymers at the end-use life cycle [3] is a desired condition required by end users in order to increase attractiveness and to diminish the environmental impact of these products.

Polylactide or polylactic acid (PLA) is one of the most promising biopolyesters considered for future developments involving the growth of global production capacities, and for the realization and commercialization of new PLA grades and PLA-based materials [4,5,6]. PLA is currently receiving considerable attention for its conventional use as a packaging material [7,8], as well as for the production of textile fibers [9] and for technical applications [10,11], if adequately modified. It has found a higher added value and remains of great interest in biomedical applications because of its biocompatibility and biodegradation/bioresorbtion [12,13].

PLA is produced from non-fossil renewable natural resources through the fermentation of polysaccharides or sugar, e.g., extracted from corn or sugar beet, and corresponding wastes, while the most relevant end-life scenario is related to its biodegradability under controlled industrial composting conditions [6] or in the natural environment [14,15] (e.g., at a lower rate in the soil). The life cycle of PLA demonstrates that this biopolyester is a performant and sustainable alternative to the petrochemical polymers with less greenhouse gas emissions, while at the end of its service life, it can be degraded to CO_2_ and biomass, allowing for a reduction in landfill volumes, thus contributing to the so-called carbon sink [14]. Moreover, the biodegradability of PLA in conjunction with the utilization of selected disposal systems, such as composting and anaerobic digestion, offers an end-of-life solution to completely remove the plastic from the environment and to close the carbon cycle [6,14].

Nowadays, the addition of selected nanofillers (e.g., organo-modified layered silicates (OMLS) [16,17], graphite/graphene derivatives [18,19,20], carbon nanotubes (CNT) [21,22,23], silver [24,25], zinc oxide (ZnO) [26,27,28], silica [29,30,31], magnetic nanoparticles [32,33,34,35,36], etc.) to PLA is considered as a modern approach that can lead to major improvements of PLA characteristics (mechanical, thermal, barrier, etc.). Furthermore, these nanocomposites could be characterized by specific end-use properties, such as anti-UV and antibacterial protection [27,37], antistatic to conductive electrical characteristics and wear resistance [1], fire-retardancy [11,18], magnetic properties [34], and so on.

Magnetic nanoparticles (NPs) from the category of iron oxides (e.g., hematite (α-Fe_2_O_3_), maghemite (γ-Fe_2_O_3_) and magnetite (Fe_3_O_4_)), due to their multiple features, including biocompatibility and low toxicity, are much more considered in the frame of academic and industrial research for a large number of applications [38]. These NPs, possessing unique biochemical, magnetic, catalytic, and other specific properties after specific modification, have proven their suitability for environmental protection [39], water purification [40], technical use (e.g., in data and energy storage, microwave absorption, and magnetic shielding [41]) and biomedical applications. Furthermore, the utilization of iron oxide NPs in biomedical applications [38,42,43,44] is of particular interest for targeted drug delivery, diagnostic magnetic resonance imaging (MRI), magnetic cell separation, tissue repair, hyperthermia, biosensors, etc. [45,46].

Magnetite is an iron oxide that exhibits very interesting properties due to the presence of both Fe(II) and Fe(III) in its structure, having particular magnetic properties that are highly valorized in biomedicine and industry [47]. It has an inverse spinel crystal structure and rare electric and magnetic features, which are based on the movements of electrons between Fe^2+^ and Fe^3+^ within the octahedral sites. Fe_3_O_4_ is considered to be one the most attractive magnetic materials, owing to its high saturation magnetization, easy synthesis, and suitable particle shape and size [48]. Furthermore, iron oxide magnetic NPs, such as magnetite nanoparticles (MNPs), are characterized by superparamagnetic properties at a low particle size (10–30 nm [47,49,50]). At such a small size, these NPs do not exhibit multiple domains as found in large magnets, whereas they behave as a single magnetic domain acting as a “single super spin”. Moreover, these NPs are characterized by negligible remanence (residual magnetization) and coercivity (the field required to bring the magnetism to zero) [51].

Unfortunately, MNPs (Fe_3_O_4_) tend to easily agglomerate under the action of van der Waals forces, while they are very sensitive to the action of moisture [52] and of oxygen from air or from water, thus they might undergo severe oxidation to ferric hydroxide (e.g., Fe(OH)_3_) or maghemite (γ-Fe_2_O_3_) [53,54]. To limit these undesired effects and/or to confer special properties to MNPs, various surface treatments and specific techniques of encapsulation are currently being considered [38,45,55,56]. The surface of MNPs can be modified to limit their aggregation and to render the nanofiller with (bio)functional groups [44,45,54]. Traditional surface treatments such as those realized with oleic acid [57], functional silanes [53,54], carboxylic acid functional poly(dimethylsiloxane) (PDMS) [58] and amino-functionalized PDMS [59], and polyvinylpyrrolidone (PVP) [60] are frequently reported in the literature. Regarding the methods to produce nanocomposites filled with MNPs, on the one hand, techniques such as solvent-casting, emulsion-evaporation, and spray drying, which involve the utilization of important amounts of organic solvents, are strongly considered at a laboratory scale to follow the constraints imposed by the biomedical application. However, according to the information given in the state of art, the iron oxide content in superparamagnetic polymer nanocomposites can be adjusted in a wide range, varying from a small weight percent up to nearly 50–60 wt.%.

On the other hand, following the increased interest in the utilization of these materials in various sectors, it is assumed by us and other researchers that the melt-compounding technique can be a more flexible and rapid method to produce polymer nanocomposites with different loadings of MNPs, having the advantages of a higher productivity, better control, and simple operation [61]. To the best of our knowledge, there are not many investigations regarding the production of PLA nanocomposites filled with MNPs, while there are a lack of studies devoted to their manufacture via melt-compounding or using the reactive extrusion (REX) approach. Furthermore, it appears that the number of research papers devoted to the effects of magnetite addition into the PLA matrix is much lower than those for other nanofillers (OMLS, CNT, graphite derivatives, halloysite nanotubes, etc.). Generally, the addition of nano- and micro-fillers into PLA can provide polymer (nano)composites with specific end-use properties, but sometimes these could have side effects such as loss of mechanical and thermal properties and degradation of the polyester matrix, aspects that need to be considered when targeting potential applications. It is worth recalling that during processing at high temperatures, PLA is particularly sensitive to the hydrolysis (e.g., the moisture can be added in the molten polyester via fillers or reinforcing fibers) [6], whereas the presence of residual catalytic metals (Sb, Zn, Fe, etc.) or metal oxides (magnetite is also included) [37,61,62] has been noted to accelerate PLA degradation by inter- and intra-molecular transesterification reactions; therefore, all precautions should be applied to limit these undesired effects.

Regarding the aims and results of this study, we would like to point out the following aspects:

(a) The first goal of this study is to propose the utilization of eco-friendly biopolymers (i.e., PLA, bio-sourced, biocompatible, and biodegradable under industrial composting conditions) for (b) the synthesis by melt-compounding (solvent-free method) of PLA nanocomposites filled with MNPs. Furthermore, (c) to render the nanofiller water repellent and (d) to limit the thermal degradation of PLA induced/catalyzed by iron (from magnetite), the effects of a novel surface treatment of MNPs have been compared with those obtained using an untreated nanofiller. 

(e) MNPs are reactively surface treated with polymethylhydrogensiloxane (MHX), followed by their dispersion into the polymer matrix using a technique (melt-compounding) that can be easy extrapolated at a larger scale, even by reactive extrusion (REX). The production of nanocomposites at a laboratory scale (micro-compounder equipment) is followed by the characterization of morphology, rheology, and of thermal properties, with a focus on the evidence of superparamagnetic characteristics. 

(f) The characterization of PLA−magnetite nanocomposites reveals significant improvements in the properties (e.g., thermal stability) when using surface treated MNPs. 

(g) Furthermore, the study is assessing the feasibility of producing superparamagnetic nanocomposites using a flexible and friendly method (melt-compounding), while it points out some questions connected to the degradation of PLA by iron at a high processing temperature, thus, using “coated” MNPs is a prerequisite.

(h) Last, but not least, we present the inherent experimental pathways connected to the production and characterization of specific features of PLA−magnetite nanocomposites, highlighting their superparamagnetic performance and their properties, making them of high interest in many applications (e.g., magnetic micro-carriers in the biomedical sector, and water purification and filtration).

## 2. Materials and Methods

### 2.1. Materials

Poly(L,L-lactide), produced and delivered as Ingeo 4032D grade (NatureWorks LLC, Blair, Nebraska, USA), was used as the polymer (PLA) matrix. The characteristics according to the information given by the supplier are as follows: relative viscosity of 3.95, D-isomer of 1.5%, and residual monomer of 0.2%.

Magnetite (supplier IOLITEC, Heilbronn, Germany) was used as the superparamagnetic nanofiller. The following characteristics are mentioned on the technical sheet: iron (II, III) oxide >98%, average particle size in the range of 20–30 nm (determined using Transmission Electron Microscopy (TEM)), specific surface area of 40–60 m^2^/g, and true density of 4.8–5.1 g/cm^3^.

XIAMETER MHX-1107 20 CST polymethylhydrogensiloxane (-Si-H as H: 1.55–1.66%; flash point (open cup): above 150 °C) [63] was kindly supplied by Dow Corning Europe (Seneffe, Belgium) and was used for the surface/reactive modification of the magnetite nanofiller. From here on it will be abbreviated as MHX.

### 2.2. Treatment of Magnetite and Production of Nanocomposites by Melt-Compounding

To produce small batches (10–20 g) of surface treated MNPs with 3% MHX, the “wet method” was primarily applied at a laboratory scale. Accordingly, the mixing and dispersion of previously dried NPs in a solution of MHX in anhydrous acetone (mechanical stirring for 60 min, 600 rpm) was followed by the removal of organic solvent and a curing process (4 h at 120 °C, under vacuum). It is also noteworthy to mention that to produce larger amounts of MNPs and nanocomposites, alternative techniques have been successfully developed by directly conducting the surface treatment of NPs with MHX in laboratory turbo-mixers (e.g., mixing 15 min at 1500 rpm).

Before processing by melt-compounding, the PLA was dried overnight at 80 °C using a drying oven with recirculating hot air. For the nanocomposite preparation, PLA (as granules) was first dry mixed with up to 16 wt.% magnetite (MNPs surface treated or not, dried under a vacuum) by shaking them into glass ampoules. The melt-compounding of blends (7 g of each composition) was performed at 190 °C (feeding for 3 min at 30 rpm, mixing in recirculating mode through the backflow channel for 10 min at 150 rpm) using a conical intermeshing corotating twin-screw micro-compounder (MiniLab II Haake Rheomex CTW5—Thermo Fischer Scientific, Karlsruhe, Germany). To gain information about the rheology/viscosity of the blends, the initial and final torque values were considered, respectively, at the beginning and the end of mixing at 150 rpm. For the sake of comparison, pristine PLA was processed under similar conditions. 

The composition and codification of the samples are shown in Table 1. Throughout this contribution, all percentages are given as weight percent (wt.%). The abbreviations M and Ms are used for untreated and surface treated MNPs, respectively.

### 2.3. Characterization Methods

#### 2.3.1. Rheological Measurements

The melt flow index (MFI) of the dried samples was determined using a Davenport 10 Melt Flow Indexer and the measurements were performed at a temperature of 190 °C following the procedure described in ASTM D1238 (standard die 2.1 mm × 8 mm and using a 2.16 kg load, Lloyd Instruments Ltd., Fareham, United Kingdom).

To gather primary information about the evolution of the melt viscosity of the blends in the micro-compounder, the initial and final torque values were recorded at the beginning and the end of mixing at 150 rpm, respectively.

#### 2.3.2. Thermogravimetric Analysis (TGA)

TGA was performed using a TGA Q50 (TA Instruments, New Castle, DE, USA) at a heating rate of 20 °C/min under N_2_ flow, from room temperature to 600 °C (platinum pan, 60 cm^3^/min nitrogen flow rate). 

Isothermal tests as simulated by TGA were performed under air (flow rate of 60 cm^3^/min). The procedure was as follows: heating rate of by 20 °C/min, from room temperature to the desired temperature (e.g., 240 °C), followed by an isothermal step for up to 60 min.

#### 2.3.3. Differential Scanning Calorimetry (DSC)

DSC measurements were performed using a DSC Q200 from TA Instruments (New Castle, DE, USA) under nitrogen flow. The procedure was as follows: first heating scan at 10 °C/min from 0 °C up to 200 °C, isotherm at this temperature for 2 min, then cooled down at 10 °C/min to −20 °C, and then a second heating scan from −20 to 200 °C at 10 °C/min. The first DSC heating scan was used to erase the prior thermal history of the samples. The events of interest, i.e., the glass transition temperature (T_g_), cold crystallization temperature(s) (T_cc_), enthalpy of cold crystallization(s) (ΔH_cc_), melting temperature (T_m_), and melting enthalpy (ΔH_m_) were typically followed in the DSC measurements. It is noteworthy to mention that in the DSC measurements for all samples, only the amount of PLA was considered, while the thermal events as evidenced by DSC in the second heating on the samples with a similar thermal history were mainly used for comparison. The degree of crystallization (see Equation (1)), all data are obtained via TA Instruments Universal Analysis 2000 software, Version 3.9A (TA Instruments—Waters LLC, New Castle, DE, USA) was determined by subtracting ΔH_cc_ from ΔH_m_ and by considering a melting enthalpy of 93 J/g for 100% crystalline PLA [28]. DSC was also used for the comparative thermal characterizations of MNPs (tests under nitrogen, rate of heating 10 °C/min).

Degree of crystallization (PLA), % = ((ΔH_m_ − (ΔH_cc1_ + ΔH_cc2_))/93) × 100 (1)

#### 2.3.4. Scanning Electron Microscopy (SEM)

SEM was performed on the samples previously cryofractured at a liquid nitrogen temperature by using a Philips XL scanning electronic microscope (Eindhoven, Netherlands) at an accelerated voltage of 20 kV and at various magnitudes. SEM was equipped for both secondary electrons (SE) and back-scattered electrons (BSE) imaging. An energy dispersive X-ray analyzer (EDX) (supplier EDAX, Tilburg, Netherlands) vas also used to provide the elemental identification of iron and oxygen from the magnetite NPs distributed through the PLA matrix.

#### 2.3.5. Transmission Electron Microscopy (TEM)

Transmission electron micrographs were obtained with a Philips CM200 (Philips, Eindhoven, Netherlands) apparatus using an accelerator voltage of up to 120 kV. Samples (70–80 nm thick) from selected PLA nanocomposites were prepared with a Leica Ultracut UCT ultracryomicrotome (Leica, Vienna, Austria) by cutting them at −100 °C.

As remark, for the investigation of the nanofillers, the surface treated or non-treated MNPs were firstly slightly pre-dispersed in acetone using a Vortex mini mixer (VWR, Leuven, Belgium). The reported microphotographs represented typical morphologies as observed at a minimum of three different locations. The measurements of the NPs size were mainly realized using ImageJ 1.52d software (National Institutes of Health, Bethesda, MD, USA) for processing and analyzing the scientific images.

#### 2.3.6. Characterization of Magnetic Properties

The measurements were performed in a DC magnetic field by means of a Quantum Design PPMS (Quantum Design, San Diego, USA) equipped with a VSM (vibrating sample magnetometer, Quantum Design, San Diego, USA) option [64]. For the measurements of the magnetization as a function of the magnetic field, i.e., of M(H), the sample was first thermally stabilized for 30 min at the measurement temperature of 300 K in the absence of the field. Then, the magnetic field was ramped to reach +9 T (1 Tesla (T) = 10^4^ Oersted (Oe)), then lowered back to −9 T, and finally increased to +9 T again, in order to acquire the complete M(H) hysteresis loops. Care was taken in the reduction of the trapped field in the magnet before starting each measurement.

## 3. Results and Discussion

### 3.1. Preliminary Considerations Regarding the Surface Treatment of Magnetite NPs with MHX

To avoid the oxidation and agglomeration of MNPs, they are usually coated during synthesis or in an additional step with organic or inorganic molecules [49]. Organofunctional silanes were considered for the modification of MNPs because they have the capability to bind to the iron oxides through covalent bonding (reaching the –OH groups at their surface) or adsorption, whereas through specific active functional groups they are able to interact with biomolecules, drugs, and metals [53]. However, MNPs need to be synthesized in an oxygen-free environment, i.e., in the presence of an inert gas, due to their high sensitivity to oxidation. Therefore, the thermal characterizations in this study were mainly performed under nitrogen atmosphere (except for the isothermal tests of nanocomposites under air).

**Scheme 1:**
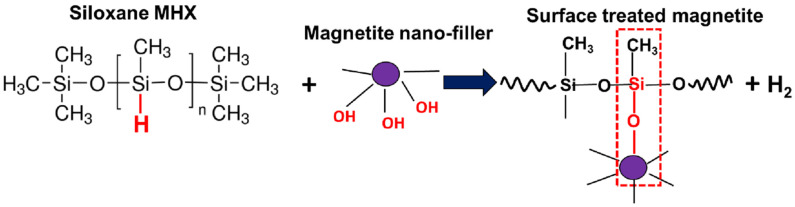
Schematic illustration of the reaction mechanism between polymethylhydrogensiloxane (MHX) and the hydroxyl groups (–OH) present at the surface of the magnetite nanofiller.

Regarding the specific surface treatment of NPs with polymethylhydrogensiloxane (MHX), through consideration of its molecular structure (i.e., a dimethyl silicone withsome methyl radicals replaced by hydrogen), each chain contained amounts of ≡Si–H (silicon hydride) and –CH_3_ (methyl) groups. Therefore, as suggested in Scheme 1, MHX was able to react at the sites of hydrogen atoms (≡Si–H) via a dehydrogenation reaction with the participation of the hydroxyl groups (–OH) present at the surface of MNPs or of other different materials (e.g., glass, leather, fabric surfaces, cellulose derivatives, and fillers such as SiO_2_), finally resulting in the covalent linkage of hydrophobic chains (MHX) onto the different substrates [65,66]. Moreover, the layers of MHX formed at the surface of MNPs could be cured by heating in the presence or absence of catalysts, with beneficial effects through developing water repellency. According to the technical sheet of the product, in a typical application, uncatalyzed films of MHX can be cured in 3 to 4 h at 120 °C, or in a shorter time, at a higher temperature [63].

**Figure 1:**
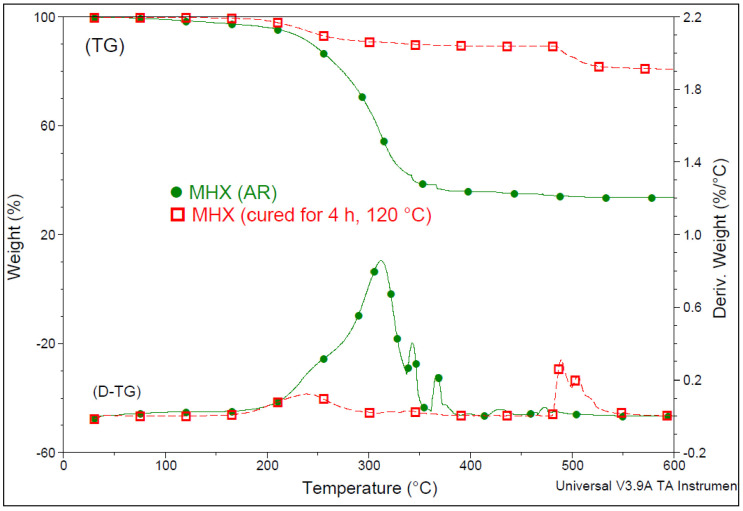
Thermogravimetric (TG) and derivative thermogravimetric (D-TG) curves of MHX with a different thermal history (under N_2_, 20 °C/min).

For better insight, Figure 1 shows the comparative TGA results of two MHX samples with a different thermal history, i.e., as received and after the curing treatment (4 h at 120 °C). Indeed, it can be seen that following the curing process, MHX became much more stable at a high temperature, thus, this feature could be advantageously valorized for the surface treatment of MNPs. Moreover, even for water-free systems, due to the presence of inherent residual moisture, the ≡Si–H bonds were hydrolyzed to ≡Si–OH, which could condense with another ≡Si–OH group (silanol condensation) or interact with ≡Si–H groups to form cross-links [67]. However, it remains to be discussed the hypothesis that in industrial conditions, as a function of the MHX percentage (in this study 3 wt.%), (a) the covalent linkage of MHX on the surface of MNPs (via –OH groups) and (b) the curing of siloxane by heating (endorsed by inherent residual moisture) [67] could occur in a simultaneous reactive process, finally leading to the coating of MNPs by water repellent siloxane layers. These aspects need more comprehension and investigation. Still, as in the case of the functionalization of (nano)fillers with silanes directly during the extrusion process (REX), it is considered that a similar procedure could also be applied for the treatment of MNPs with MHX.

**Figure 2:**
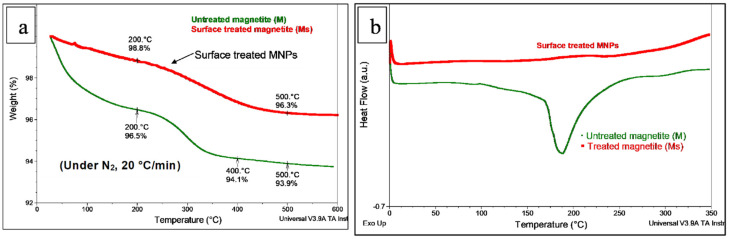
(**a**,**b**) Comparative (**a**) TG and (**b**) DSC curves of treated (Ms) and untreated (M) magnetite NPs.

By comparing the MNPs tested in the frame of the study (i.e., untreated (M) and surface treated (Ms)) through both TGA and DSC analyses (Figure 2a,b), it can be seen that following treatment with MHX, the NPs of magnetite became more stable, i.e., less sensitive to the absorption of moisture. According to TGA (Figure 2a), the weight losses of the superficial moisture and physisorbed (chemisorbed) water molecules up to a temperature of 200 °C for M and Ms were 3.5% and 1.2%, respectively. On the other hand, a large endotherm was evidenced by DSC for the untreated magnetite (peak at about 190 °C), whereas this thermal event was not evidenced in the case of MNPs treated with MHX. This endotherm is traditionally ascribed in the literature to the removal of free and physisorbed water from MNPs [52], but we did not exclude the influence of other factors/thermal events that could occur concurrently, such as the presence of traces of iron hydroxide (FeO(OH) and/or Fe(OH)_3_, and their dehydration to ferric oxide [68,69]), or of some residual products, such as metallic impurities or surfactants of fatty acids [70].

**Figure 3:**
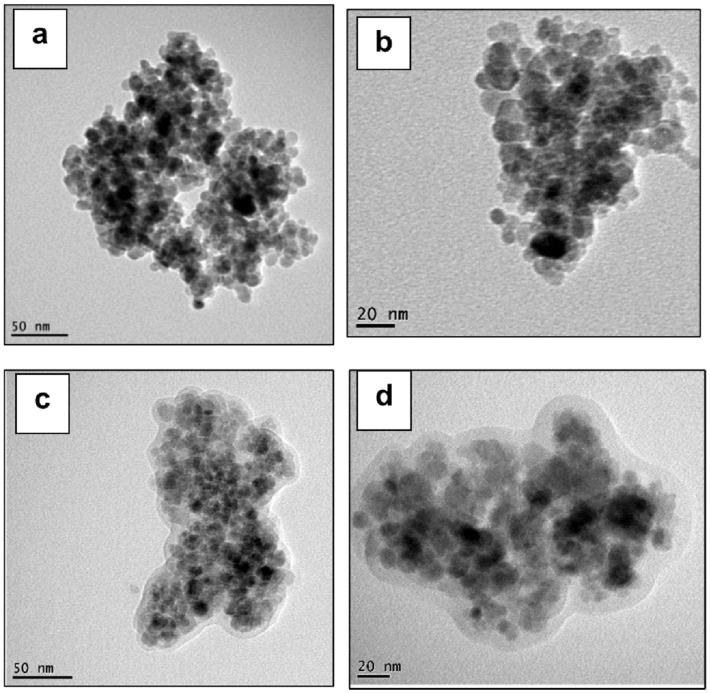
(**a**–**d**) TEM pictures of untreated magnetite NPs (**a**,**b**) and NPs surface treated with MHX (**c**,**d**).

Regarding the morphology of MNPs, Figure 3a–d shows selected TEM pictures of NPs, before and after treatment with MHX. The nanofiller is mostly as aggregates composed of spherical-like primary particles of a low dimension (typically 10–20 nm). 

**Figure 4:**
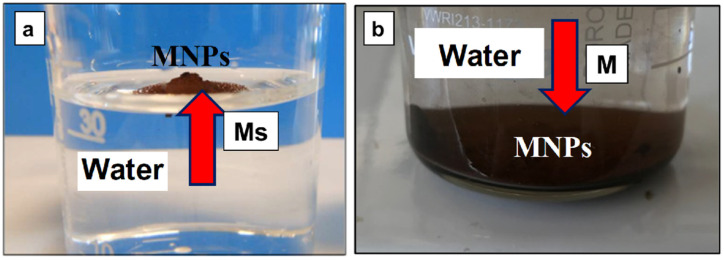
(**a**,**b**) Evidence of the properties of hydrophobicity following the immersion in water of (**a**) surface treated magnetite with MHX compared with (**b**) the pristine nanofiller (hydrophilic). Abbreviations: MNPs = magnetite nanoparticles; M and Ms are the symbols for untreated and surface treated magnetite nanofiller, respectively.

Following surface treatment, the NPs were coated/encapsulated within a network of polysiloxane chains (Figure 3c,d), which effectively led to the separation and limitation of interactions between MNPs. On the other hand, as shown in Figure 4a,b, following a test of dispersion in water, it is clearly seen that the treated MNPs became hydrophobic due to the presence of siloxane layers at their surface when compared with the untreated nanofiller (M), which assessed the hydrophilic properties. However, for the production of PLA-magnetite nanocomposites at a larger scale, the specific surface treatment of MNPs requires optimization and additional characterizations via different techniques, not developed in this paper (Fourier transform infrared spectroscopy (FTIR), X-ray photoelectron spectroscopy (XPS), contact angle measurements, etc.). Evidence of whether the coatings of different natures will modify (decrease) sensitivity to the oxidation of magnetite NPs is still of interest.

### 3.2. Characterization of PLA-Magnetite Nanocomposites

#### 3.2.1. Effects of Magnetite Addition and MNPs Surface Treatment on PLA Thermal Properties

As stated in the Introduction, the addition of nanofillers into PLA could provide polymer nanocomposites with specific end-use properties, but there could also sometimes be undesired effects, such as the loss of mechanical and thermal properties due to the advanced degradation of the polyester matrix [6]. Regarding the thermal characteristics, the addition of NPs into PLA can lead to opposite effects, i.e., increasing or diminishing of PLA thermal stability [1,37]. It was revealed elsewhere [61,62] that the presence of metal oxides in PLA (the iron oxides are included) is detrimental to the thermal stability of the polymer matrix. Moreover, the occurrence of residual catalytic metals (Sb, Zn, Al, Fe, etc.) in PLA has been noted to accelerate inter- and intra-molecular transesterification reactions at a high temperature.

**Figure 5:**
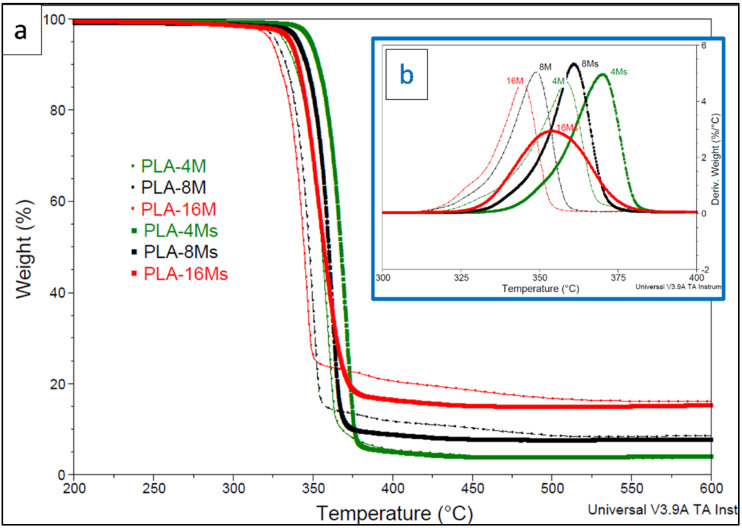
(**a**,**b**) Comparative thermogravimetric traces ((**a**)TG and (**b**) D-TG, in the inset of the figure) of PLA−magnetite nanocomposites filled with 4–16% untreated (M) and surface treated (Ms) magnetite nanoparticles (under nitrogen, 20 °C/min).

Figure 5a,b shows a direct comparison of the thermogravimetric (TG (a)) and derivative thermogravimetric (D-TG (b)) traces obtained following the heating of PLA-magnetite nanocomposites containing amounts of nanofiller (surface treated or not) ranging from 4% to 16% MNPs under nitrogen. First, it is worth pointing out that the decrease in the thermal stability of PLA nanocomposites (ascribed to the catalytic effects of iron) is clearly determined by the loading of magnetite, while in all cases, it has seen a beneficial boosting effect following surface treatment with MHX. Still, for better insight, Table 2 shows the values of the temperatures corresponding to 5% weight loss and to the maximum rate of degradation (T_5%_ and T_D_, respectively, from the derivative thermogravimetric curves (D-TG)). T_5%_ is often considered as the onset of the decomposition temperature, while the residue at 600 °C gives information about the amounts of inorganic filler in the final nanocomposites. 

By increasing the percentage of untreated nanofiller (M) in PLA up to 16 wt.%, the onset of thermal degradation decreased gradually by up to 30 °C compared with the neat PLA (T_5%_ of 354 °C). Still, at different magnetite amounts, the values of T_D_ followed similar tendencies, with the most important drop being of about 43 °C in the case of the PLA-16M sample (T_D_ = 344 °C compared with those of PLA (T_D_ = 387 °C)). The important degradation effects of iron on PLA macromolecular chains are explained by the high capacity of transition metals to coordinate ester groups and to accelerate the transesterification and depolymerization reactions at a high temperature [62]. On the other hand, it is noteworthy to mention that in all cases, the utilization of surface treated magnetite (Ms) led to better stability and improved thermal characteristics. The values of both T_5%_ and T_D_ were significantly higher compared with those of the nanocomposites containing similar amounts of untreated magnetite. These improvements are reasonably ascribed to the presence of polysiloxane layers at the interface between NPs and the polyester matrix, with beneficial effects for retarding the catalytic effects of iron on PLA. Thus, as in the case of MgO and ZnO, nanofillers well recognized for the strong catalytic action on PLA [37], these results show that the surface treatment of MNPs is a precondition to limit the catalytic effect of iron, which is determined by the loading of magnetite. 

**Figure 6:**
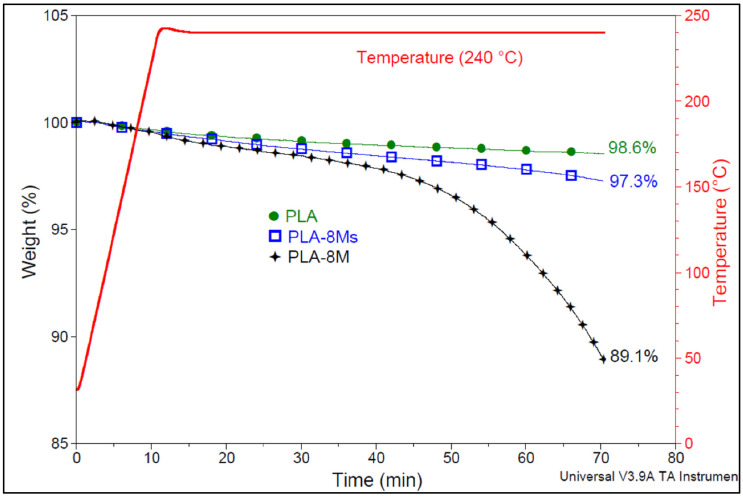
Comparative isothermal tests under air at 240 °C for PLA, PLA-8M, and PLA-8M nanocomposites.

Considering that the maximal temperature of PLA processing is about 240 °C, Figure 6 shows the comparative behavior of neat PLA and of selected nanocomposites (PLA-8Ms and PLA-8M) at this temperature under isothermal conditions (under air, with a residence time of up to 60 min). The neat PLA shows the best thermal stability, with only 1.4 wt.% loss. However, it is obvious that the catalytic effect of iron (from Fe_3_O_4_) is much more reduced in the case of surface treated MNPs (Ms), i.e., the weight loss is only 2.7%, whereas in the case of nanocomposites containing untreated nanofiller (M), it is much more impressive (i.e., about 10.9%).

**Figure 7:**
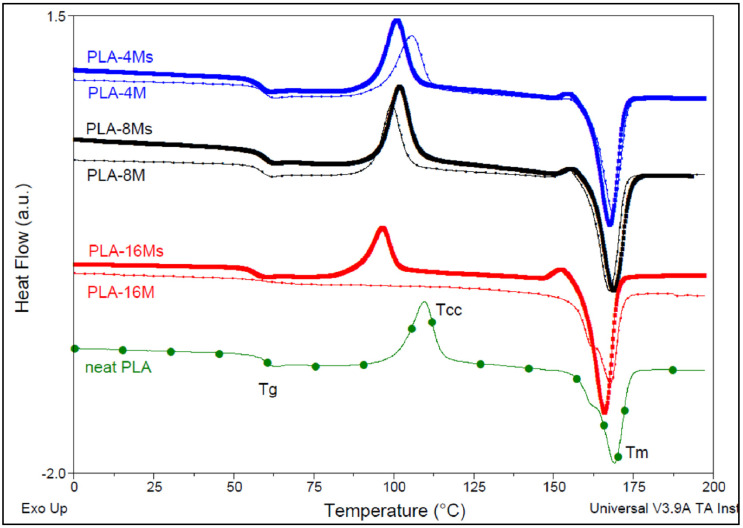
Comparative DSC traces of neat PLA and of PLA–magnetite nanocomposites with similar loadings of nanofiller (second DSC heating, 10 °C/min).

In relation to the thermal parameters as evaluated by the DSC analysis (Table 3, Figure 7), from the results obtained for nanocomposites with a similar thermal history, it can be seen that the addition and increase of nanofiller (M and Ms) loading led to the slight diminishing of the glass transition temperatures (T_g_) when compared with the neat PLA, behavior that could be reasonably be ascribed to the formation of PLA chains of lower molecular weights (see also the Section 3.2.2.) due to polyester degradation at high shearing during the melt-compounding process. However, the influence of other factors, such as the properties of lubrication assigned to the presence of MNPs [71], is not totally excluded. Regarding the values of the main peak of cold crystallization (T_cc1_), the crystallization of PLA at a lower temperature is particularly evidenced at higher nanofiller loading in the case of nanocomposites containing both untreated (M) and surface treated nanofiller (Ms). On the other hand, it is noteworthy to mention that a great part of nanocomposites (all samples containing Ms) showed a second small exothermic peak (T_cc2_) just before melting, which has traditionally been ascribed to the transformation of disordered α′ crystals to the ordered α-form in the literature [72]. Supplementary Materials S1 shows additional information obtained using the DSC analysis, i.e., the comparative DSC traces acquired during the cooling and subsequent (second) heating (10 °C/min) of PLA–magnetite nanocomposites produced with untreated and surface treated magnetite nanoparticles (Figure S1a,b). Still, from the information obtained via the DSC analysis, it can be seen that after adding high amounts of MNPs (i.e., 16%), there was a spectacular effect on the rate of crystallization of PLA, with effects on the final degree of crystallinity, with values of about 40.1% and 24.9% being obtained through the addition of 16% M and Ms, respectively. Nevertheless, the thermal events evidenced by DSC could be influenced by concurrent factors, such as the formation of products of lower molecular weights induced by the shear stress during melt-compounding, the catalytic effect of iron oxide, and degradation due to the inherent moisture, among others. Furthermore, the nature of the PLA matrix (content of D-isomer) and its molecular weights, percentage of nanofiller, specific processing conditions, and so on, are parameters that require careful consideration when are compared the results of different studies [61].

#### 3.2.2. Rheological Characterizations

**Figure 8:**
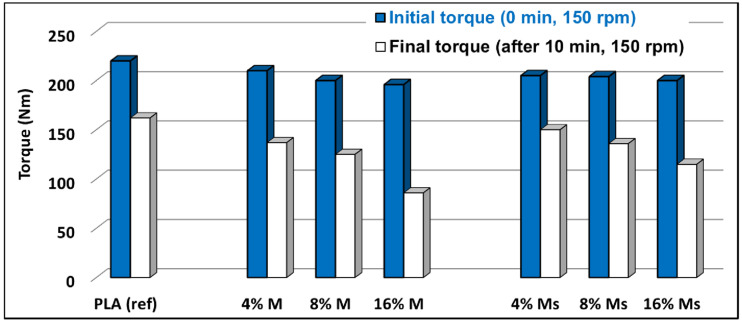
Evolution of torque values during melt-mixing of PLA and of PLA−magnetite nanocomposites.

It is noteworthy to mention that the micro-compounder used for the realization of nanocomposites was built with the ability to give rheological information during melt-compounding (torque of the motor, drop of pressures in the backflow channel, values of temperature, etc.). By measuring the torque and the pressure in the backflow channel, the compounding process is monitored effectively. The evolution and values of torque during melt-mixing at similar speeds for the twin-screws are conventionally correlated with the viscosity of blends in the molten state. Figure 8 shows the comparative evolution of the torque values (i.e., at the beginning and at the end of melt-compounding by 150 rpm) of neat PLA and of PLA-magnetite nanocomposites at similar shear rates. As it is usually expected, in the initial phase of melting/mixing, the differences between the behavior of PLA and PLA-nanocomposites was less significant. Nevertheless, following the melt-compounding process, the viscosity of the samples in the molten state decreased in correlation with the residence time under a high shear stress. At the end of melt-mixing, the decrease of torque became evident for all PLA samples, and this was even more important in the case of the nanocomposites containing untreated nanofiller (M). However, in comparison with the pristine PLA, the reduction of torque was quite well determined by the loading of nanofiller, whereas an eventual lubricant effect conferred by siloxane (MHX) was less evident. 

On the other hand, similar tendencies regarding the melt rheology of samples were evidenced by the determination of MFI values (Table 4), which confirmed the increase of melt fluidity in the following order: PLA < PLA–xMs < PLA-xM. However, the increased fluidity could be reasonably ascribed to the decrease of PLA molecular masses due to shear, the presence of nanofiller, and inherent residual moisture, all with potential degradation effects, without excluding the influence of other factors. Nevertheless, the drop of melt viscosity due to the degradation of PLA in the presence of magnetite was evidenced elsewhere by performing dynamic rheological characterizations, and the complex viscosity of PLA−magnetite composites was found to be lower than that of neat PLA [61]. It is also noteworthy to remember that both magnetite [71] and siloxanes (PDMS) are credited with lubricating properties; thus, the accurate interpretation of the rheology of nanocomposites becomes more difficult when taking into account the occurrence of different factors. Unfortunately, the attempts to characterize the nanocomposites through size exclusion chromatography (SEC) and to obtain information about the changes of PLA molecular weights were not reproducible enough due to difficulties in the separation of nanofillers (NPs of 10–20 nm); thus, this aspect, which is apparently missing in the literature, must be considered in a forthcoming study.

#### 3.2.3. Morphology of PLA−Magnetite Nanocomposites

To reveal the morphology of PLA−magnetite nanocomposites, different techniques of microscopy (SEM in SE and BSE mode, SEM-EDX (energy-dispersive X-ray spectroscopy), and TEM on chosen specimens) were considered to characterize selected samples produced in the frame of this study. 

**Figure 9:**
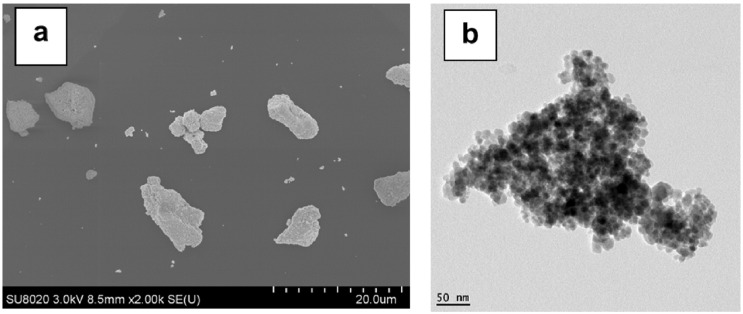
(**a**,**b**) SEM (**a**) and TEM (**b**) images of magnetite NPs revealing that the primary nanoparticles are agglomerates.

First, it was considered of interest to show the comparative images of the nanofillers as they were obtained using both SEM and TEM methods (Figure 9a,b, respectively). It can be seen that the information obtained via the two techniques was very different, whereas, once more, the primary nanoparticles of magnetite are noted to be agglomerates.

**Figure 10:**
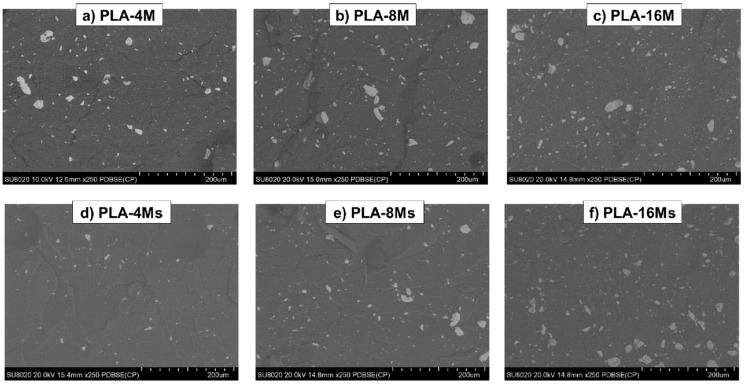
(**a**–**f**) SEM pictures of PLA–magnetite nanocomposites produced using untreated magnetite (**a**–**c**) and surface treated nanofiller (**d**–**f**).

Regarding the morphology of the nanocomposites, the SEM analysis in SE mode (NB: with secondary electrons) gave only initial information about the topography of the cryofractured surfaces, but this procedure was considered less helpful to obtain accurate insight regarding the distribution of magnetite nanofiller (Supplementary Materials S1, and Figure S2a,b). Fortunately, backscattered-electron (BSE) imaging was found to give a clearer indication about the presence or lack of aggregates/clusters of NPs in nanocomposites, their dimension, and their distribution in the superficial zone of the samples. Figure 10a–f shows selected SEM (BSE) micrographs of PLA–magnetite nanocomposites with different loadings of non-treated and treated MNPs. The presence of clusters of MNPs was better evidenced, especially for the nanocomposites containing non-treated magnetite (Figure 10a–c), and more specifically, for increased amounts of nanofiller (8–16 wt.%). However, at high loadings of surface treated magnetite (Ms), clusters of magnetite NPs were also present. From the SEM-BSE micrographs, which give partial information at a microscale level, it is difficult to conclude that at high filling, the surface treatment of MNPs allowed for significant improvements regarding the morphology of nanocomposites, taking into account the tendency of particles of a low dimension to form aggregates as well as the limits of micro-compounder equipment. 

**Figure 11:**
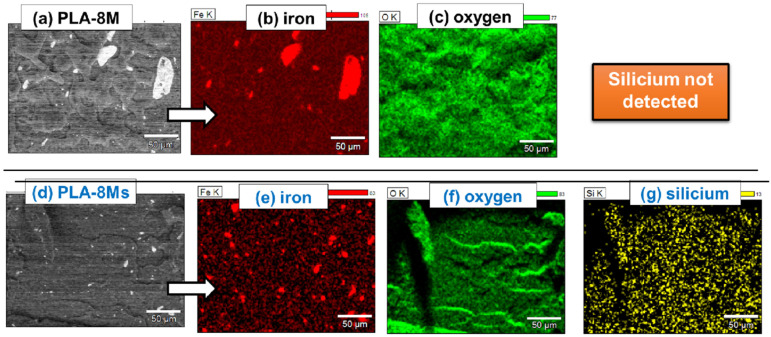
(**a**–**g**) SEM and EDX elemental mapping on PLA-8M (**a**–**c**) and PLA-8Ms nanocomposites (**d**–**g**) to evidence the presence of oxygen and iron as the main elements from magnetite nanofiller (presence of Si in PLA-8Ms (Figure 11g) is due to the treatment of NPs with MHX).

Nevertheless, better evidence regarding the distribution of nanofillers is obtained via elemental EDX mapping of Fe and O as the main elements from MNPs (Fe_3_O_4_) and of Si from MHX (Ms) in the case of nanocomposites containing treated nanofiller (Figure 11a–g). These elements are not present only in the clusters identified in SEM-BSE micrographs, but also as NPs finely distributed/dispersed within the PLA matrix. EDX analysis did not evidence any traces of Si in the case of nanocomposites filled with untreated nanofiller. However, the TEM analysis (on selected samples) was once more proven to be a very powerful technique for obtaining information regarding the dispersion of NPs at a nanoscale. 

**Figure 12:**
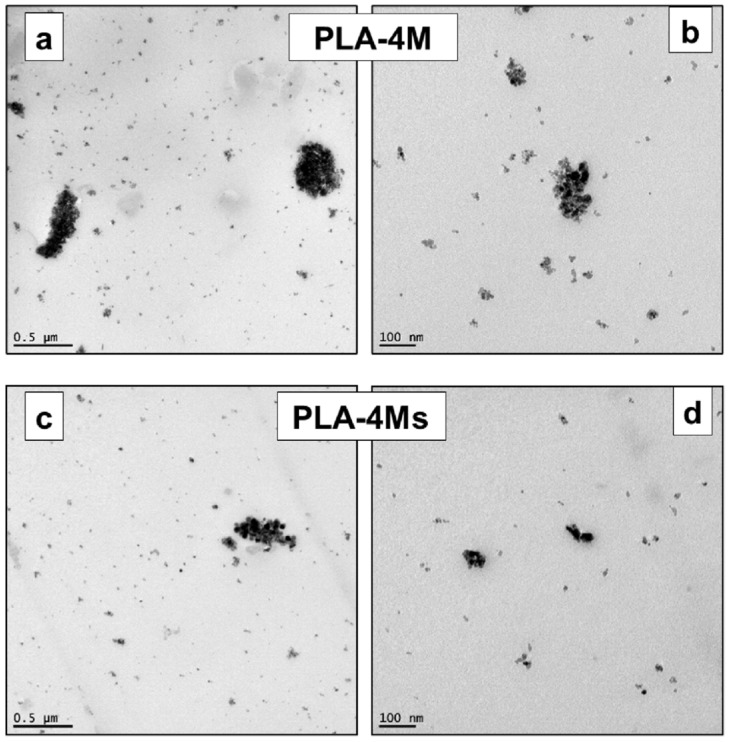
(**a**–**c**) TEM pictures at different magnifications of PLA–4% magnetite nanocomposites produced using untreated (**a**,**b**) and surface treated MNPs (**c**,**d**).

Figure 12a–d shows representative TEM pictures of the PLA nanocomposites filled with 4% MNPs (surface treated and non-treated). In agreement with the conclusion of SEM-BSE analyses, the clusters of bigger dimensions were found, to some extent, to be more frequent in the case of samples containing untreated nanofillers. Furthermore, in all pictures performed at a high magnification, the presence of isolated NPs and of associations/clusters composed by few MNPs appeared to be more evident. Nevertheless, it is assumed that a better distributive/dispersive mixing will certainly be obtained in the case of the laboratory/industrial extrusion equipment recognized with an enhanced melt-blending capability. Still, the fine distribution/dispersion of MNPs of low dimensions into a polymer of a high viscosity can be a very complex process. Indeed, even at a lower percentage of magnetite (less than 5 wt.%), based on SEM analysis, it was elsewhere reported that the presence of “sea-island” structures of MNPs are distributed through the PLA matrix [61]. Therefore, alternative/optimized techniques and enhanced melt-mixing conditions are of further interest.

#### 3.2.4. Magnetic Features of PLA–Magnetite Nanocomposites

**Figure 13:**
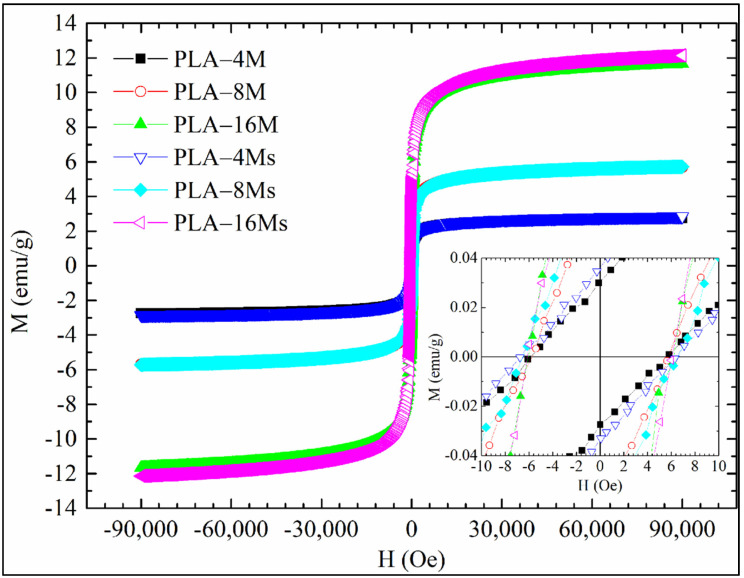
M(H) at a temperature of 300 K of PLA nanocomposites filled with 4–16% untreated and surface treated magnetite nanoparticles.

**Figure 14:**
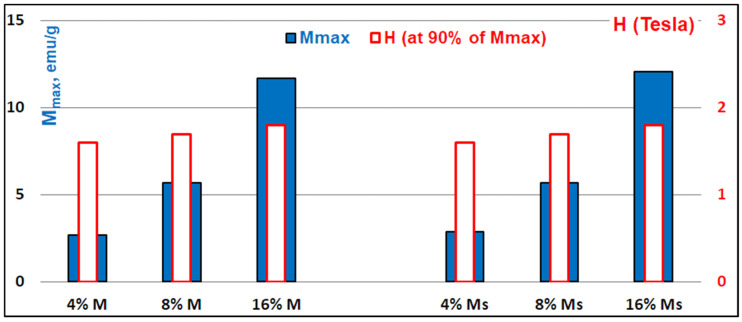
Comparative magnetic features of PLA−magnetite nanocomposites filled with 4–16% untreated (M) and surface treated (Ms) magnetite nanoparticles: M_max_ and H at 90% of M_max_.

MNPs are typically in a superparamagnetic state at room temperature if their diameters are extremely low. The level of magnetization can be saturated under a specific external field, whereas in its absence, the net magnetic moments are often randomized to zero [34]. Based on the literature data, the magnetization values for superparamagnetic iron oxide nanoparticles (SPIONs) range from 30 to 60 emu/g, while higher values (i.e., about 90 emu/g) have been observed for bulk materials [46,73]. The magnetization (M) curves as a function of the magnetic field (H) of PLA nanocomposites containing both untreated and surface treated MNPs, are shown in Figure 13, whereas in Figure 14, their specific magnetic features are compared (i.e., the maximum magnetization (M_max_) and H at 90% of M_max_).

It is worth noting that, for each sample, the full magnetic saturation (Ms) was not reached at the maximum applied field (i.e., H = 9 T; 1 T = 10^4^ Oe), while 90% of the maximum magnetization (M_max_) at 9 T was reached for a magnetic field (H) in the range 1.6 T–1.8 T. 

The magnetization of nanocomposites is increasing promptly with the applied magnetic field towards the maximum magnetization state (M_max_) determined by the loading of MNPs, while when the magnetic field is decreased, the magnetization curves follow the same pathways by passing through the initial starting points, just with a negligible hysteretic behavior. As it is expected, the maximum magnetization signal in nanocomposites increased in very good correlation with the magnetite amounts [74], with the samples filled with 16% MNPs being characterized by the highest response rate. It is also important to specify that the inset from Figure 13 shows the M(H) curves near a zero magnetic field. It can be noted that for all of the samples there was a very low coercive field equal to ± 6 Oe, whereas at similar nanofiller loading, the treatment of MNPs with MHX did not significantly modify the magnetic behavior of the samples.

Despite the presence of some clusters of MNPs evidenced by SEM analysis, the magnetic coercivity (H_c_) of nanocomposite samples (±6 Oe) was experimentally found to be lower than the theoretical value ascribed to superparamagnetism (H_c_ ≤ 50 Oe) [75]. As for all samples H_c_ had very low values, the superparamagnetic properties of the nanocomposites were well confirmed. For more information regarding the magnetic features of PLA−magnetite nanocomposites produced using untreated nanofiller, we also recommend a recent study published by us [64]. 

In the view of the biomedical applications, lower magnetic fields could be required (e.g., 0.2 T) [76]. Interestingly, at 0.2 T magnetic field (H), all samples attained about 68% of M_max_. Furthermore, the magnetic characterizations did not evidence some differences that could be attributed to the effects of surface treatments, while to obtain the magnetic saturation (M_sat_) level, higher magnetic fields are required (i.e., >9 T).

#### 3.2.5. PLA–Magnetite Nanocomposites with Superparamagnetic Properties: Current Prospects

From the experimental results of this study, in which the equipment for melt-mixing was a laboratory micro-compounder that only allowed the production of small quantities of material characterized by superparamagnetic properties, the up-scaling on bigger laboratory twin-screw extruders and the tailoring of properties of PLA−magnetite nanocomposites remains of current interest. Regarding potential applications, it is noteworthy to mention that these nanocomposites could be designed for utilization in various sectors, from biomedical to technical applications. In biomedical applications, adequately biofunctionalized and/or modified magnetite/nanocomposites with suitable surface chemistry could be used as magnetic carriers for cell separation systems, as biosensors, as magnetic-resonance image (MRI) contrast agents, etc., while many technical sectors could valorize their magnetic features (water purification, microwave absorption, magnetic shielding, etc.). 

**Figure 15:**
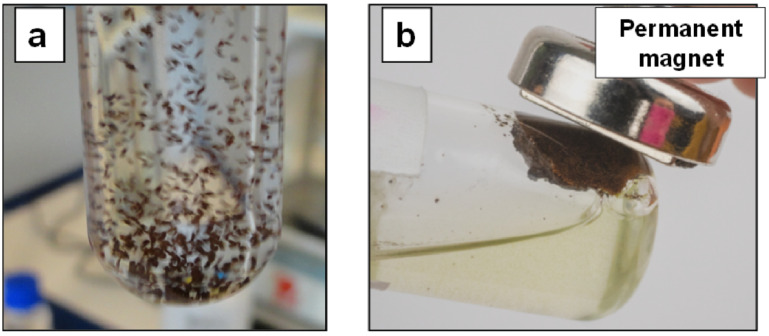
(**a**,**b**) Dispersion of PLA−magnetite nanocomposites (microparticles) in PBS (**a**) and illustration of magnetic separation (**b**).

Figure 15a,b illustrates the principle of magnetic separation (one key application) using the superparamagnetic properties of new materials in a potential application as microcarriers, e.g., for the production of stem cells [77]. The dispersion of microparticles of PLA−magnetite nanocomposites (obtained by cryogenic grinding) in a selected media (e.g., PBS (phosphate buffered saline)), is followed by their easy separation/recovering in a magnetic field. 

Finally, it is assumed that the melt-compounding/REX is a very flexible technique for the production of nanocomposites filled with MNPs, whereas, on the other hand, the realization of highly filled masterbatches can also be an option of interest. Still, the utilization of eco-friendly materials for applications which need superparamagnetic properties can represent an alternative of choice for the partial substitution of traditional polymers of a petrochemical origin (PS, PMMA, etc.); thus, forthcoming studies and additional characterizations (e.g., for the evaluation of mechanical properties, to confirm the stability of nanocomposites under different conditions of processing and utilization) will be required to extrapolate the PLA−magnetite nanocomposites of interest at a larger scale.

## 4. Conclusions

To propose the utilization of eco-friendly materials characterized by superparamagnetic properties, this study focused on the effects of the addition of magnetite nanoparticles (MNPs) in the PLA matrix (bio-sourced polyester, biocompatible, and biodegradable). Magnetite with NPs of low dimensions (10–20 nm) were considered as superparamagnetic nanofillers to produce PLA nanocomposites. Furthermore, the surface treatment of NPs with reactive polymethylhydrogensiloxane (MHX) was considered for making the nanofiller hydrophobic, less sensitive to the action of moisture, and to reduce the catalytic effects at the high processing temperature of iron (from magnetite) on the polyester matrix. PLA was mixed by melt-compounding with 4–16 wt.% magnetite NPs (surface treated or non-treated) using a laboratory micro-compounder, following a traditional melt-blending technique which does not require any solvent. 

The thermal characterizations revealed significant improvements in the thermal stability of nanocomposites using surface treated NPs (M_s_) by performing both non-isothermal and isothermal TGAs. Accordingly, it was concluded that the presence polysiloxane layers on the surface of NPs has a key role in retarding the catalytic effects of iron from magnetite (Fe_3_O_4_) on polyester (PLA) macromolecular chains. From the DSC characterizations, a spectacular increase in the degree of crystallization of PLA was evidenced, especially at higher amounts of MNPs (i.e., 16 wt.%), ascribed to occurrent factors (formation of products of lower molecular weights due to shear, catalytic effect of iron oxide, others). Compared with the neat PLA, the decrease of melt viscosity (increase of fluidity in MFI rheological measurements) was more important at high loading of MNPs and using untreated nanofillers. Moreover, different techniques of microscopy (SEM, EDX, and TEM) have been used to reveal the morphology of nanocomposites. Together with well distributed/dispersed NPs (evidenced via SEM and EDX analysis), the presence of clusters (somewhat more frequent at higher magnetite loadings, and in the case of non-treated MNPs) was ascribed to the aptitude of MNPs to form aggregates, without excluding some inherent limits connected to the micro-compounder equipment. 

The VSM measurements of PLA−magnetite nanocomposites evidenced their superparamagnetic behavior, with near zero magnetic remanence and coercivity, and strong magnetization properties even at low magnetic field (H = 0.2 T). However, the maximum magnetization signal (M_max_) was found to be directly linked to the loading of nanofillers, without significant influence connected to the surface treatment of NPs. By considering the flexibility of melt-compounding (REX) techniques, these nanocomposites could be further optimized and proposed as an alternative for the partial substitution of petrochemical polymers, for utilization in similar applications. The production of performant PLA nanocomposites characterized by superparamagnetic properties could pave the way to the larger utilization of biopolymers in technical and biomedical sectors (e.g., as superparamagnetic micro-carriers).

## Figures and Tables

**Table 1:** Composition (wt.%) and codification of nanocomposite samples obtained by melt compounding.

Sample	Code	PLA	Nanofiller, wt.%	
		wt.%	Untreated	Treated *
PLA−4% magnetite	PLA-4M	96	4	-
PLA−8% magnetite	PLA-8M	92	8	-
PLA−16% magnetite	PLA-16M	84	16	-
PLA−4% treated magnetite *	PLA-4Ms	96	-	4
PLA−8% treated magnetite	PLA-8Ms	92	-	8
PLA−16% treated magnetite	PLA-16Ms	84	-	16

* Surface-treated MNPs with 3% MHX.

**Table 2:** Onset of thermal degradation (T_5%_), decomposition temperature (T_D_), and residual products of PLA and PLA–magnetite nanocomposites as determined by TGA (under nitrogen, 20 °C/min).

Sample	T_5%_ Temperature at 5% Weight Loss, °C	T_D_ Temperature at Max. Rate of Degradation, °C	Residual Product at 600 °C, %
Neat PLA	354	387	0.3
PLA-4M	332	358	3.8
PLA-4Ms	349	370	4.1
PLA-8M	327	349	8.6
PLA-8Ms	341	361	7.7
PLA-16M	324	344	16.1
PLA-16Ms	336	354	15.2

**Table 3:** Comparative DSC data of PLA and PLA-magnetite nanocomposites (second DSC heating, 10 °C/min).

Sample	T_g_ (°C)	T_cc1_ (°C)	ΔH_cc1_ (J g^−1^)	T_cc2_ (°C)	ΔH_cc2_ (J g^−1^)	T_m_ (°C)	ΔH_m_ (J g^−1^)	χ_c,_ %
PLA	61	109	24.8	-	-	169	36.7	12.8
PLA-4M	60	106	27.3	-	-	168	39.5	13.1
PLA-8M	59	99	23.4	155	1.6	168	38.3	14.3
PLA-16M	58	-	-	-	-	167	37.3	40.1
PLA-4Ms	59	101	26.9	151	0.5	167	41.5	15.2
PLA-8Ms	60	102	27.7	151	1.1	168	40.9	13.0
PLA-16Ms	57	96	17.4	152	1.8	166	42.4	24.9

Abbreviations: glass transition temperature (T_g_), cold crystallization temperature (T_cc_), enthalpy of cold crystallization (ΔH_cc_), melting temperature (T_m_), melting enthalpy (ΔH_m_), degree of crystallization (χ_c,_).

**Table 4:** Characterization of melt-flow index (MFI; 190 °C, 2.16 kg) of PLA and PLA−magnetite nanocomposites.

Sample	MFI (g/10 min)
PLA (AR)	3.1
PLA (processed)	5.8
PLA-4M	9.2
PLA-8M	10.8
PLA-16M	14.9
PLA-4Ms	7.8
PLA-8Ms	8.7
PLA-16Ms	10.6

## Data Availability

Data available in a publicly accessible repository: Pieces of information connected to this study have been presented as poster communication and are available as supporting material on the ResearchGate website page of M.M. at the following web address (https://www.researchgate.net/publication/331950866_Pathways_to_green_perspective_Polylactide_PLA_nanocomposites_with_superparamagnetic_properties). Data citation: Murariu, M.; Galluzzi, A.; Raquez, J.-M.; Polichetti, M. and Dubois, Ph., *Pathways to green perspective: Polylactide (PLA) nanocomposites with superparamagnetic properties*, international conference “*Which sustainable future for plastic*?”, 21 March 2019, Mons, Belgium, DOI: 10.13140/RG.2.2.32119.47522.

## Supplementary Materials

The following are available online at https://www.mdpi.com/article/10.3390/ma14185154/s1, Figure S1a,b Comparative DSC traces obtained during cooling and subsequent (second) heating (10 °C/min) of PLA–magnetite nanocomposites produced using untreated (a) and surface treated magnetite nanoparticles (b), and Figure S2a,b SEM pictures in SE mode of PLA—8% magnetite nanocomposites (PLA-8M and PLA-8Ms) produced using untreated (a) and surface treated magnetite (b).

## References

[1] Raquez J.-M., Habibi Y., Murariu M., Dubois P. (2013). Polylactide (PLA)-based nanocomposites. Prog. Polym. Sci..

[2] Babu R.P., O’Connor K., Seeram R. (2013). Current progress on bio-based polymers and their future trends. Prog. Biomater..

[3] Rezvani Ghomi E., Khosravi F., Saedi Ardahaei A., Dai Y., Neisiany R.E., Foroughi F., Wu M., Das O., Ramakrishna S. (2021). The life cycle assessment for polylactic acid (PLA) to make it a low-carbon material. Polymers.

[4] Sreekumar K., Bindhu B., Veluraja K. (2021). Perspectives of polylactic acid from structure to applications. Polym. Renew. Resour..

[5] Banerjee R., Ray S.S. (2021). An overview of the recent advances in polylactide-based sustainable nanocomposites. Polym. Eng. Sci..

[6] Murariu M., Dubois P. (2016). PLA composites: From production to properties. Adv. Drug Deliv. Rev..

[7] Auras R., Harte B., Selke S. (2004). An overview of polylactides as packaging materials. Macromol. Biosci..

[8] Widiastuti I. (2016). Polylactide nanocomposites for packaging materials: A review. AIP Conf. Proc..

[9] Gupta B., Revagade N., Hilborn J. (2007). Poly(lactic acid) fiber: An overview. Prog. Polym. Sci..

[10] Nagarajan V., Mohanty A.K., Misra M. (2016). Perspective on polylactic acid (PLA) based sustainable materials for durable applications: Focus on toughness and heat resistance. ACS Sustain. Chem. Eng..

[11] Murariu M., Laoutid F., Dubois P., Fontaine G., Bourbigot S., Devaux E., Campagne C., Ferreira M., Solarski S. (2014). Chapter 21—Pathways to biodegradable flame retardant polymer (nano)composites. Polymer Green Flame Retardants.

[12] Saini P., Arora M., Kumar M.N.V.R. (2016). Poly(lactic acid) blends in biomedical applications. Adv. Drug Deliv. Rev..

[13] Lasprilla A.J.R., Martinez G.A.R., Lunelli B.H., Jardini A.L., Filho R.M. (2012). Poly-lactic acid synthesis for application in biomedical devices—A review. Biotechnol. Adv..

[14] Teixeira S., Eblagon K.M., Miranda F., Pereira M.F.R., Figueiredo J.L. (2021). Towards controlled degradation of poly(lactic) acid in technical applications. C J. Carbon Res..

[15] Ncube L.K., Ude A.U., Ogunmuyiwa E.N., Zulkifli R., Beas I.N. (2020). Environmental impact of food packaging materials: A review of contemporary development from conventional plastics to polylactic acid based materials. Materials.

[16] Sinha Ray S., Maiti P., Okamoto M., Yamada K., Ueda K. (2002). New polylactide/layered silicate nanocomposites. 1. Preparation, characterization, and properties. Macromolecules.

[17] Okamoto M., Avérous L., Pollet E. (2012). Polylactide/clay nano-biocomposites. Environmental Silicate Nano-Biocomposites.

[18] Murariu M., Dechief A.L., Bonnaud L., Paint Y., Gallos A., Fontaine G., Bourbigot S., Dubois P. (2010). The production and properties of polylactide composites filled with expanded graphite. Polym. Degrad. Stab..

[19] Kim I.-H., Jeong Y.G. (2010). Polylactide/exfoliated graphite nanocomposites with enhanced thermal stability, mechanical modulus, and electrical conductivity. J. Polym. Sci. Part B Polym. Phys..

[20] Wang Y., Lin C.-S. (2013). Preparation and characterization of maleated polylactide-functionalized graphite oxide nanocomposites. J. Polym. Res..

[21] Pillai S.K., Ramontja J., Ray S.S. (2011). Amine functionalization of carbon nanotubes for the preparation of CNT based polylactide composites—A comparative study. Nanostructured Materials and Nanotechnology V.

[22] Wu D., Lv Q., Feng S., Chen J., Chen Y., Qiu Y., Yao X. (2015). Polylactide composite foams containing carbon nanotubes and carbon black: Synergistic effect of filler on electrical conductivity. Carbon.

[23] Brzeziński M., Biela T. (2014). Polylactide nanocomposites with functionalized carbon nanotubes and their stereocomplexes: A focused review. Mater. Lett..

[24] Shameli K., Ahmad M.B., Wan Yunus W.M.Z., Ibrahim N.A., Jokar M., Darroudi M. (2010). Synthesis and characterization of silver/polylactide nanocomposites. World Acad. Sci. Eng. Technol..

[25] Kim E.S., Kim S.H., Lee C.H. (2010). Electrospinning of polylactide fibers containing silver nanoparticles. Macromol. Res..

[26] Chu Z., Zhao T., Li L., Fan J., Qin Y. (2017). Characterization of antimicrobial poly (lactic acid)/nano-composite films with silver and zinc oxide nanoparticles. Materials.

[27] Marra A., Silvestre C., Duraccio D., Cimmino S. (2016). Polylactic acid/zinc oxide biocomposite films for food packaging application. Int. J. Biol. Macromol..

[28] Murariu M., Benali S., Paint Y., Dechief A.-L., Murariu O., Raquez J.-M., Dubois P. (2021). Adding value in production of multifunctional polylactide (PLA)–ZnO nanocomposite films through alternative manufacturing methods. Molecules.

[29] Zhu A., Diao H., Rong Q., Cai A. (2010). Preparation and properties of polylactide–silica nanocomposites. J. Appl. Polym. Sci..

[30] Huang J.-W., Chang Hung Y., Wen Y.-L., Kang C.-C., Yeh M.-Y. (2009). Polylactide/nano and microscale silica composite films. I. Preparation and characterization. J. Appl. Polym. Sci..

[31] Odent J., Raquez J.-M., Samuel C., Barrau S., Enotiadis A., Dubois P., Giannelis E.P. (2017). Shape-memory behavior of polylactide/silica ionic hybrids. Macromolecules.

[32] Nan A., Turcu R., Liebscher J. (2012). Magnetite-polylactic acid core–shell nanoparticles by ring-opening polymerization under microwave irradiation. J. Polym. Sci. Part A Polym. Chem..

[33] Chen A.-Z., Kang Y.-Q., Pu X.-M., Yin G.-F., Li Y., Hu J.-Y. (2009). Development of Fe_3_O_4_-poly(l-lactide) magnetic microparticles in supercritical CO_2_. J. Colloid Interface Sci..

[34] Zhang X., Xue L., Wang J., Liu Q., Liu J., Gao Z., Yang W. (2013). Effects of surface modification on the properties of magnetic nanoparticles/PLA composite drug carriers and in vitro controlled release study. Colloids Surf. A Physicochem. Eng. Asp..

[35] Shan D., Shi Y., Duan S., Wei Y., Cai Q., Yang X. (2013). Electrospun magnetic poly(l-lactide) (PLLA) nanofibers by incorporating PLLA-stabilized Fe_3_O_4_ nanoparticles. Mater. Sci. Eng. C.

[36] Yang G., Zhang B., Wang J., Wang M., Xie S., Li X. (2016). Synthesis and characterization of poly(lactic acid)-modified superparamagnetic iron oxide nanoparticles. J. Sol-Gel Sci. Technol..

[37] Murariu M., Doumbia A., Bonnaud L., Dechief A.L., Paint Y., Ferreira M., Campagne C., Devaux E., Dubois P. (2011). High-performance polylactide/ZnO nanocomposites designed for films and fibers with special end-use properties. Biomacromolecules.

[38] Natarajan S., Harini K., Gajula G.P., Sarmento B., Neves-Petersen M.T., Thiagarajan V. (2019). Multifunctional magnetic iron oxide nanoparticles: Diverse synthetic approaches, surface modifications, cytotoxicity towards biomedical and industrial applications. BMC Mater..

[39] Kalia S., Kango S., Kumar A., Haldorai Y., Kumari B., Kumar R. (2014). Magnetic polymer nanocomposites for environmental and biomedical applications. Colloid Polym. Sci..

[40] Martinez-Boubeta C., Simeonidis K., Thomas S., Pasquini D., Leu S.-Y., Gopakumar D.A. (2019). Chapter 20—Magnetic Nanoparticles for Water purification. Nanoscale Materials in Water Purification.

[41] Frey N.A., Peng S., Cheng K., Sun S. (2009). Magnetic nanoparticles: Synthesis, functionalization, and applications in bioimaging and magnetic energy storage. Chem. Soc. Rev..

[42] Wu W., Wu Z., Yu T., Jiang C., Kim W.-S. (2015). Recent progress on magnetic iron oxide nanoparticles: Synthesis, surface functional strategies and biomedical applications. Sci. Technol. Adv. Mater..

[43] Wu W., He Q., Jiang C. (2008). Magnetic iron oxide nanoparticles: Synthesis and surface functionalization strategies. Nanoscale Res. Lett..

[44] Demirer G.S., Okur A.C., Kizilel S. (2015). Synthesis and design of biologically inspired biocompatible iron oxide nanoparticles for biomedical applications. J. Mater. Chem. B.

[45] Oh J.K., Park J.M. (2011). Iron oxide-based superparamagnetic polymeric nanomaterials: Design, preparation, and biomedical application. Prog. Polym. Sci..

[46] Kandasamy G., Maity D. (2015). Recent advances in superparamagnetic iron oxide nanoparticles (SPIONs) for in vitro and in vivo cancer nanotheranostics. Int. J. Pharm..

[47] Li Q., Kartikowati C.W., Horie S., Ogi T., Iwaki T., Okuyama K. (2017). Correlation between particle size/domain structure and magnetic properties of highly crystalline Fe_3_O_4_ nanoparticles. Sci. Rep..

[48] Lu Q., Choi K., Nam J.-D., Choi H.J. (2021). Magnetic polymer composite particles: Design and magnetorheology. Polymers.

[49] Ali A., Zafar H., Zia M., Ul Haq I., Phull A.R., Ali J.S., Hussain A. (2016). Synthesis, characterization, applications, and challenges of iron oxide nanoparticles. Nanotechnol. Sci. Appl..

[50] Baumgartner J., Bertinetti L., Widdrat M., Hirt A.M., Faivre D. (2013). Formation of magnetite nanoparticles at low temperature: From superparamagnetic to stable single domain particles. PLoS ONE.

[51] Wahajuddin S.A. (2012). Superparamagnetic iron oxide nanoparticles: Magnetic nanoplatforms as drug carriers. Int. J. Nanomed..

[52] Baharvand H. (2008). Encapsulation of ferromagnetic iron oxide particles by polyester resin. e-Polymers.

[53] Yamaura M., Camilo R.L., Sampaio L.C., Macêdo M.A., Nakamura M., Toma H.E. (2004). Preparation and characterization of (3-aminopropyl)triethoxysilane-coated magnetite nanoparticles. J. Magn. Magn. Mater..

[54] Bini R.A., Marques R.F.C., Santos F.J., Chaker J.A., Jafelicci M. (2012). Synthesis and functionalization of magnetite nanoparticles with different amino-functional alkoxysilanes. J. Magn. Magn. Mater..

[55] Aisida S.O., Akpa P.A., Ahmad I., Zhao T.-K., Maaza M., Ezema F.I. (2020). Bio-inspired encapsulation and functionalization of iron oxide nanoparticles for biomedical applications. Eur. Polym. J..

[56] Akbarzadeh A., Samiei M., Davaran S. (2012). Magnetic Fe_3_O_4_: Preparation, physical properties, and applications in biomedicine. Nanoscale Res. Lett..

[57] Frounchi M., Shamshiri S. (2015). Magnetic nanoparticles-loaded PLA/PEG microspheres as drug carriers. J. Biomed. Mater. Res. Part A.

[58] Wilson K.S., Goff J.D., Riffle J.S., Harris L.A., St Pierre T.G. (2005). Polydimethylsiloxane-magnetite nanoparticle complexes and dispersions in polysiloxane carrier fluids. Polym. Adv. Technol..

[59] de Rezende Bonesio M., Pissetti F.L. (2020). Magnetite particles covered by amino-functionalized poly(dimethylsiloxane) network for copper(ii) adsorption from aqueous solution. J. Sol-Gel Sci. Technol..

[60] Pandey G., Singh S., Hitkari G. (2018). Synthesis and characterization of polyvinyl pyrrolidone (PVP)-coated Fe_3_O_4_ nanoparticles by chemical co-precipitation method and removal of congo red dye by adsorption process. Int. Nano Lett..

[61] Yu B., Wang M., Sun H., Zhu F., Han J., Bhat G. (2017). Preparation and properties of poly (lactic acid)/magnetic Fe_3_O_4_ composites and nonwovens. RSC Adv..

[62] Cam D., Marucci M. (1997). Influence of residual monomers and metals on poly (l-lactide) thermal stability. Polymer.

[63] Xiameter™ MHX-1107 Fluid 20cst. https://www.Dow.Com/en-us/pdp.Xiameter-mhx-1107-fluid-30-cst.04088544h.Html.

[64] Galluzzi A., Murariu M., Raquez J.-M., Polichetti M., Dubois P. (2021). Effect of magnetite nanoparticles content on the magnetic properties of polylactide and polystyrene composites. Chem. Eng. Trans..

[65] Lin H., Hu Q., Liao T., Zhang X., Yang W., Cai S. (2020). Highly hydrophobic cotton fabrics modified by poly(methylhydrogen)siloxane and fluorinated olefin: Characterization and applications. Polymers.

[66] Chen J., Ma Y., Lin H., Zheng Q., Zhang X., Yang W., Li R. (2019). Fabrication of hydrophobic ZnO/PMHS coatings on bamboo surfaces: The synergistic effect of ZnO and PMHS on anti-mildew properties. Coatings.

[67] Roy Choudhury A.K. (2017). Principles of Textile Finishing.

[68] Khatavkar S.N., Sartale S.D. (2017). α-Fe_2_O_3_ thin films by liquid phase deposition: Low-cost option for supercapacitor. J. Solid State Electrochem..

[69] Taghizadeh S.-M., Berenjian A., Zare M., Ebrahiminezhad A. (2020). New perspectives on iron-based macrostructure. Processes.

[70] Kim D.K., Lee J.W. (2018). Synthesis of non-hydrate iron oleate for eco-friendly production of monodispersed iron oxide nanoparticles. J. Korean Ceram. Soc..

[71] Gao C., Wang Y., Hu D., Pan Z., Xiang L. (2013). Tribological properties of magnetite nanoparticles with various morphologies as lubricating additives. J. Nanopart. Res..

[72] Saeidlou S., Huneault M.A., Li H., Park C.B. (2012). Poly(lactic acid) crystallization. Prog. Polym. Sci..

[73] Namanga J., Foba J., Ndinteh D.T., Yufanyi D.M., Krause R.W.M. (2013). Synthesis and magnetic properties of a superparamagnetic nanocomposite “pectin-magnetite nanocomposite”. J. Nanomater..

[74] Di Palma L., Bavasso I., Sarasini F., Tirillò J., Puglia D., Dominici F., Torre L., Galluzzi A., Polichetti M., Ramazanov M.A. (2018). Effect of nano-magnetite particle content on mechanical, thermal and magnetic properties of polypropylene composites. Polym. Compos..

[75] Jiang H., Han X., Li Z., Chen X., Hou Y., Gai L., Li D., Lu X., Fu T. (2012). Superparamagnetic core–shell structured microspheres carrying carboxyl groups as adsorbents for purification of genomic DNA. Colloids Surf. A Physicochem. Eng. Asp..

[76] Pankhurst Q.A., Connolly J., Jones S.K., Dobson J. (2003). Applications of magnetic nanoparticles in biomedicine. J. Phys. D: Appl. Phys..

[77] Tavassoli H., Alhosseini S.N., Tay A., Chan P.P.Y., Weng Oh S.K., Warkiani M.E. (2018). Large-scale production of stem cells utilizing microcarriers: A biomaterials engineering perspective from academic research to commercialized products. Biomaterials.

