# Supplementary material for: Pathways to Green Perspectives: Production and Characterization of Polylactide (PLA) Nanocomposites Filled with Superparamagnetic Magnetite Nanoparticles
Source: Materials (Basel). 2021 Sep 8;14(18):5154. doi: 10.3390/ma14185154 (PMC8467987; doi:10.3390/ma14185154)
Supplement: Supplementary file 1 [file materials-14-05154-s001.zip › materials-1335575-supplementary.pdf]

Supplementary materials

Pathways to Green Perspectives: Production and Characterization of Polylactide (PLA) Nanocomposites Filled with Superparamagnetic Magnetite Nanoparticles

Marius Murariu [1,*], Armando Galluzzi [2], Yoann Paint [1], Oltea Murariu [1], Jean-Marie Raquez [3], Massimiliano Polichetti [2] and Philippe Dubois [1,3,*]

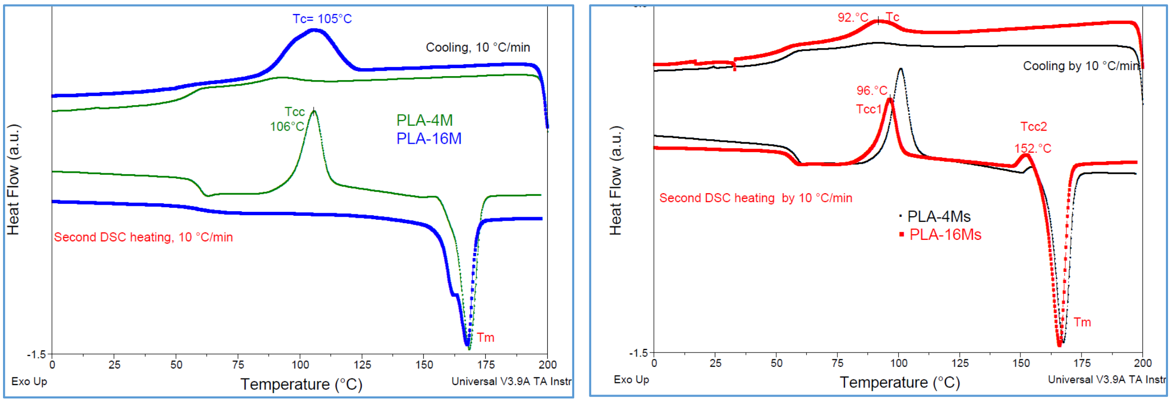

(**a**) (**b**)

Figure S1. (**a**,**b**). Comparative DSC traces obtained during cooling and subsequent (second) heating (10 °C/min) of PLA–magnetite nanocomposites produced using untreated (**a**) and surface treated magnetite nanoparticles (**b**).

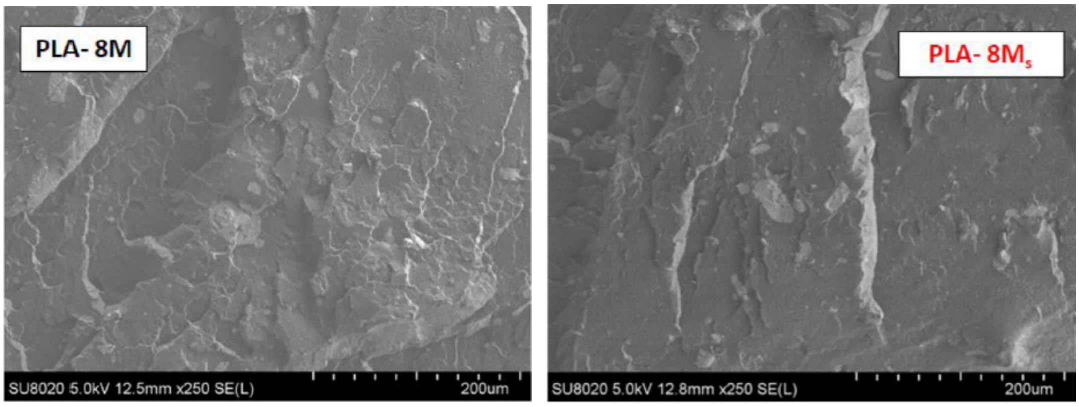

(**a**) (**b**)

Figure S2. (**a**,**b**) SEM pictures in SE mode of PLA—8% magnetite nanocomposites (PLA-8M and PLA-8Ms) produced using untreated (**a**) and surface treated magnetite (**b**).
